# Supplementary material for: Epidemiological, Clinical and Genetic Features of ALS in the Last Decade: A Prospective Population-Based Study in the Emilia Romagna Region of Italy
Source: Biomedicines. 2022 Mar 31;10(4):819. doi: 10.3390/biomedicines10040819 (PMC9031824; doi:10.3390/biomedicines10040819)
Supplement: Supplementary file 1 [file biomedicines-10-00819-s001.zip › biomedicines-1589600-supplementary.pdf]

Supplementary material

ERRALS group
Project coordinator: Dr J. Mandrioli.
Collaborating centers:
Department of Neurosciences, Azienda Ospedaliero Universitaria di Modena and Department of Biomedical, Metabolic and Neural Sciences, University of Modena and Reggio Emilia, Modena, Italy (Jessica Mandrioli, Nicola Fini, Ilaria Martinelli, Elisabetta Zucchi, Giulia Gianferrari, Cecilia Simonini, Annalisa Gessani, Stefano Meletti, Marco Vinceti);
Dipartimento di Scienze Biomediche e Neuromotorie, University of Bologna, and IRCCS Istituto delle Scienze Neurologiche di Bologna, Bellaria Hospital, Bologna, Italy (Veria Vacchiano and Rocco Liguori);
IRCCS Istituto delle Scienze Neurologiche di Bologna, Bellaria Hospital, Bologna, Italy (Fabrizio Salvi, Ilaria Bartolomei and Roberto Michelucci);
Dipartimento di Scienze Biomediche e Neuromotorie, University of Bologna, Bologna (Pietro Cortelli);
IRCCS Istituto delle Scienze Neurologiche di Bologna, Department of Neurology and Stroke Center, Maggiore Hospital, Bologna, Italy (Anna Maria Borghi, Andrea Zini)
IRCCS Istituto delle Scienze Neurologiche di Bologna, UOC Interaziendale Clinica Neurologica Metropolitana (NeuroMet), Bologna, Italy (Rita Rinaldi and Pietro Cortelli);
Department of Neurosciences and Rehabilitation, St Anna Hospital, Ferrara (Elisabetta Sette, Valeria Tugnoli);
Department of Neuroscience and Rehabilitation, University of Ferrara, Ferrara (Maura Pugliatti);
Department of Neurology, IRCCS Arcispedale Santa Maria Nuova, Reggio Emilia (Elena Canali, Luca Codeluppi and Franco Valzania);
Department of Neuroscience, University of Parma, Parma, Italy (Lucia Zinno, Salvatore Stano and Giovanni Pavesi);
Department of Neurology, Fidenza Hospital, Parma (Doriana Medici and Giovanna Pilurzi);
Department of Neurology, G. Da Saliceto Hospital, Piacenza (Emilio Terlizzi and Donata Guidetti);
Department of Neurology, Carpi Hospital, Modena (Silvia De Pasqua and Mario Santangelo);
Department of Neurology, Imola Hospital, Bologna (Martina Bracaglia and Patrizia De Massis);
Department of Neurology, Faenza and Ravenna Hospital, Ravenna (Mario Casmiro and Pietro Querzani);
Department of Neurology, Bufalini Hospital, Cesena (Simonetta Morresi, Maria Vitiello and Marco Longoni);
Department of Neurology, Forlì Hospital, Forlì (Alberto Patuelli, Susanna Malagù, Francesca Bianchi and Marco Longoni);
Department of Neurology, Infermi Hospital, Rimini (Marco Currò Dossi, Cristiana Ganino and Marco Longoni);
Department of Hospital Services, Emilia Romagna Regional Health Authority, Bologna (Salvatore Ferro).
